# Vitamin B12 Deficiency in Persons with Intellectual Disability in a Vegetarian Residential Care Community

**DOI:** 10.1100/tsw.2005.11

**Published:** 2005-01-21

**Authors:** Mohammed Morad, Mark Gringols, Isack Kandel, Joav Merrick

**Affiliations:** ^1^ Kfar Rafael Residential Care Center, Clalit Health Services and Division for Community Health, Ben Gurion University of the Negev, Beer-Sheva, Israel; ^2^ Clalit Health Services and Recanati School for Community Health Professions, Faculty of Health Sciences, Ben Gurion University of the Negev, Beer-Sheva, Israel; ^3^ Faculty of Social Science, Department of Behavioral Sciences, Academic College of Judea and Samaria, Ariel, Israel; ^4^ National Institute of Child Health and Human Development, Office of the Medical Director, Division for Mental Retardation, Ministry of Social Affairs, Jerusalem and Zusman Child Development Center, Division of Pediatrics and Community Health, Ben Gurion University, Beer-Sheva, Israel

**Keywords:** cobalamin, vitamin B12 deficiency, prevalence, intellectual disability, mental retardation, residential care, Israel

## Abstract

The goal of this study was to determine the prevalence of vitamin B12 deficiency among intellectually disabled persons in a vegetarian remedial community in Israel. In this community, 47 individuals with intellectual disability (ID) live in 7 enlarged families in a kibbutz style agricultural setting. These 47 individuals and 17 of their caregivers were screened for vitamin B12 deficiency. There were 25.5% of the disabled vs. 11.8% of the caregivers found to have levels of vitamin B12 lower than 157 pg/ml. It is concluded that persons with ID in this vegetarian residential care community seemed to be at a higher risk for vitamin B12 deficiency.

## INTRODUCTION

The prevalence of vitamin B12 (cobalamin) deficiency can range from 3–29%[1] in the general population, whereas in vegetarians it can reach up to 40%[2]. The daily intake of vitamin B12 in the Western diet is 5–15 mcg/day[3] and the intake in individuals with intellectual disability (ID) has been measured to be 10.8 mcg daily[4]. The minimum daily requirement for cobalamin is 2.5 mcg according to Harrison's Principles of Internal Medicine[5].

Since persons with ID also live in households, communities, or residential care centers with a vegetarian nutritional way of life, the literature was surveyed to find information on vitamin B12 deficiency, but the prevalence of vitamin B12 deficiency in this population has not been studied before. The purpose of the present study was therefore to determine the prevalence of vitamin B12 or cobalamin deficiency in persons with ID and their caregivers in a vegetarian remedial community.

## METHODS

The Division for Mental Retardation in Israel provides service to over 25,000 persons with ID and among them, 6,500 individuals in close to 60 residential care centers around the country. One center in the south of Israel was selected for this pilot study. This center has been arranged like a kibbutz with seven housing units, each with a family, their own children, and seven persons with ID, who share living quarters, kitchen, and dining room. During the day, the family members, other staff, volunteers, and the people with ID work together in the fields, a bread factory, an olive oil press, a wax candle factory, and a weaving factory.

The inspiration for this therapeutic approach came from the German-Austrian philosopher Rudolf Steiner (1861–1925), and today there are about 300 therapeutic communities around the world working with persons with ID at all ages. In all these communities, an effort is made to harmonize the different elements of life, because the basic problem is considered to be a “dissonance” within the “symphony of life” and the healing process tries to find a suitable “consonant” solution. Work, social life, and cultural life serve as therapeutic activities together with individual treatment. The more harmonious the development of the person, the more independent the person becomes in achieving harmony in the environment. A vegetarian lifestyle is part of the therapeutic philosophy.

As part of the yearly routine blood examination of the residents with ID, a specimen was drawn for vitamin B12 levels and examined at the Clalit Health Service Laboratory (normal range 157–1059 pg/ml). In addition, 17 vegetarian caregivers were examined. Statistical analysis was not found to be relevant in this small study.

## RESULTS

The residential care center studied had 47 adults (22 females and 25 males): 42 persons aged 19–45 years, 4 aged 46–60 years, and 1 above 61 years. In addition, 11 had mild, 26 moderate, and 10 severe ID.

Of the 47 persons with ID screened, 12/47 (25.5%) were found to have vitamin B12 levels lower than 157 pg/ml, while 2/17 (11.8%) of the caregivers group had vitamin B12 lower than 157 pg/ml.

Looking at the vitamin B12 levels in persons with ID from various etiologies, persons with ID and cerebral palsy had the lowest average level (200 pg/ml), followed by fragile X (206.7 pg/ml), while persons with Down syndrome had the highest average (272 pg/ml). Persons with epilepsy had lower vitamin B12 deficiency (average at 180.72 pg/ml) than persons without epilepsy (average at 260.02 pg/ml).

## DISCUSSION

There are very few studies on vitamin B12 deficiency in the population of persons with ID. Cunningham and co-workers from Stewarts Hospital in Ireland[4] studied the dietary intake of 115 male and 217 female persons with mental handicap (aged 15–64 years) in five long-term institutions and found the vitamin B12 intake to be 10.8 mcg/day.

Hanley et al.[6] (from the Hospital for Sick Children in Toronto) described a case of an 18-year-old female with phenylketonuria (PKU) who presented at follow-up after years of absence with various symptoms such as spastic paraparesis, tremor, disorientation, slurred speech, distractibility, deteriorating mental function, and megaloblastic anemia. During the investigation, vitamin B12 deficiency was found and after treatment, most of her symptoms disappeared. The case story resulted in further investigation of 37 adolescents at that clinic with PKU and it was found that 6 (16%) had subnormal serum B12 levels and another 6 had borderline low values. Another case story[7] of an adult with PKU and vitamin B12 deficiency has been reported.

We have not been able to find studies of vitamin B12 deficiency in the population of vegetarians with ID, but Pongstaporn and Bunyaratavej[2] studied 179 vegetarians and 58 controls without ID in Thailand. They found that hemoglobin, hematocrit, mean corpuscular hemoglobin, mean corpuscular hemoglobin concentration, white blood cells, neutrophils, serum ferritin, and serum vitamin B12 in vegetarians were significantly lower than controls ($p < 0.05$). Vitamin B12 deficiency was found in 40% of the vegetarians.

Our small sample showed that a large number (25.5%) of the persons with ID living on a vegetarian diet and 11.8% of their caregivers living in the same environment and eating the same food have vitamin B12 deficiency. Since the dietary source of cobalamin is animal products, such as meat and dairy foods, it is no wonder that vegetarians are prone to deficiency.

## CONCLUSION

It seems that persons with ID in this residential care center are at a higher risk for vitamin B12 deficiency. We therefore recommend routine investigations for vitamin B12 levels in this population.

## BIOSKETCHES

**Mohammed Morad, MD**, is a specialist in family medicine, lecturer in family medicine at the National Institute of Child Health and Human Development, Division of Community Health, Ben Gurion University of the Negev, and the medical director of a large area clinic in the city of Beer-Sheva. Publications include those on Bedouin health, health aspects, spiritual health, and aging in persons with intellectual disability, and he is a presenter on topics like health policy and services for disadvantaged at national and international conferences. E-mail: morad62@barak-online.net

**Mark Gringols, BPT, MPH,** is a physiotherapist educated at the Department of Physical Therapy, School for Allied Health Professions, Sackler School of Medicine, Tel Aviv University and Department of Epidemiology, Division of Public Health, Faculty of Health Sciences, Ben Gurion University of the Negev, Beer-Sheva, Israel. He works as a clinical physiotherapist for the Clalit Health Services in Beer-Sheva and as a clinical instructor at the Recanati School for Community Health Professions, Faculty of Health Sciences, Ben Gurion University of the Negev, Beer-Sheva for students in physiotherapy during their practical clinical period in the community. E-mail: gringols@bezeqint.net

**Isack Kandel**, **MA, PhD**, is senior lecturer at the Faculty of Social Sciences, Department of Behavioral Sciences, the Academic College of Judea and Samaria, Ariel. During the period 1985–1993, he served as the director of the Division for Mental Retardation, Ministry of Social Affairs, Jerusalem, Israel. E-mail: Kandeli@aquanet.co.il

**Joav Merrick, MD, DMSc,** is professor of child health and human development affiliated with the Zusman Child Development Center, Division of Pediatrics and Community Health at the Ben Gurion University, Beer-Sheva, Israel; the medical director of the Division for Mental Retardation, Ministry of Social Affairs, Jerusalem; and founder and director of the National Institute of Child Health and Human Development. He has numerous publications in the field of child health and human development, rehabilitation, intellectual disability, disability, health, welfare, abuse, advocacy, quality of life and prevention. Dr. Merrick received the Peter Sabroe Child Award for outstanding work on behalf of Danish Children in 1985 and the International LEGO-Prize ("The Children's Nobel Prize") for an extraordinary contribution towards improvement in child welfare and well being in 1987. E-mail: jmerrick@internet-zahav.net. Website: www.nichd-israel.com
